# The Caregiver Initiative: A Model for Caregiver Health and Wellness in Age-Friendly Health Systems

**DOI:** 10.1093/geroni/igab046.1030

**Published:** 2021-12-17

**Authors:** Ellen Carbonell, Robyn Golden, Leslie Pelton

**Affiliations:** 1 Rush University Medical Center, Chicago, Illinois, United States; 2 Institute for Healthcare Improvement, Boston, Massachusetts, United States

## Abstract

Health care systems have historically relied on family caregivers to provide long-term care for older adults, often including medically complex care for which they have no medical training. Yet there are few interventions in place to assist them within health care systems. The Rush Caregiver Initiative (Rush-CGI) provides system-level and caregiver interventions. Rush-CGI’s system-level interventions focus on culture change, including modifying workflows, training, and electronic health record data. Rush-CGI caregiver interventions begin with assessment of needs and provision of resources for caregivers, offering interprofessional family-based interventions. Interventions include a Teach-Back Clinic, Family Care Planning sessions, and Goals of Medical Care meetings, all held on an outpatient basis either in person or virtually. Outcomes include decreased caregiver depression and anxiety, and increased caregiving self-efficacy. This presentation will discuss creating system level change and providing customized caregiver interventions, including how the Rush-CGI can be modified to fit a variety of patient populations.

